# Two unexpected promiscuous activities of the iron–sulfur protein IspH in production of isoprene and isoamylene

**DOI:** 10.1186/s12934-016-0476-9

**Published:** 2016-05-11

**Authors:** Deyong Ge, Yanfen Xue, Yanhe Ma

**Affiliations:** State Key Laboratory of Microbial Resources, Institute of Microbiology, Chinese Academy of Sciences, Beijing, 100101 People’s Republic of China; University of Chinese Academy of Sciences, Beijing, 100049 People’s Republic of China; College of Medicine, Anhui University of Science and Technology, Huainan, People’s Republic of China

**Keywords:** *Bacillus*, Isoprene, 2-Methyl-2-butene, 3-Methyl-1-butene, (*E*)-4-Hydroxy-3-methyl-2-butenyl diphosphate (HMBPP), Dimethylallyl diphosphate (DMAPP), Isoprene synthase, IspS, IspH

## Abstract

**Background:**

*Bacillus* species, possessing the methylerythritol phosphate (MEP) pathway for the synthesis of isoprenoid feedstock, are the highest producers of isoprene among bacteria; however, the enzyme responsible for isoprene synthesis has not been identified. The iron–sulfur protein IspH is the final enzyme of the MEP pathway and catalyses the reductive dehydration of (*E*)-4-hydroxy-3-methyl-2-butenyl diphosphate (HMBPP) to form isopentenyl diphosphate and dimethylallyl diphosphate (DMAPP). In this study, we demonstrated two unexpected promiscuous activities of IspH from alkaliphilic *Bacillus* sp. N16-5, which can produce high levels of isoprene.

**Results:**

*Bacillus* sp. N16-5 IspH could catalyse the formation of isoprene from HMBPP and the conversion of DMAPP into a mixture of 2-methyl-2-butene and 3-methyl-1-butene. Both reactions require an electron transfer system, such as that used for HMBPP dehydration. Isoprene and isoamylene synthesis in *Bacillus* sp. N16-5 was investigated and the reaction system was reconstituted in vitro, including IspH, ferredoxin and ferredoxin-NADP^+^-reductase proteins and NADPH. The roles of specific IspH protein residues were also investigated by site-directed mutagenesis experiments; two variants (H131N and E133Q) were found to have lost the HMBPP reductase activity but could still catalyse the formation of isoprene. Overexpression of IspH H131N in *Bacillus* sp. N16-5 resulted in a twofold enhancement of isoprene production, and the yield of isoprene from the strain expressing E133Q was increased 300 % compared with the wild-type strain.

**Conclusions:**

IspH from *Bacillus* sp. N16-5 is a promiscuous enzyme that can catalyse formation of isoprene and isoamylene. This enzyme, especially the H131N and E133Q variants, could be used for the production of isoprene from HMBPP.

**Electronic supplementary material:**

The online version of this article (doi:10.1186/s12934-016-0476-9) contains supplementary material, which is available to authorized users.

## Background

Isoprene and isoamylene are important platform chemicals in the synthetic chemistry industry. There are very few organisms which can produce isoamylene, while isoprene is one of the most abundant natural products in the environment and can be produced naturally by a wide variety of organisms, including animals, plants, and bacteria [1–3]. Isoprene production in plants has been well studied, and isoprene synthase (IspS) has been identified from several plants, such as kudzu and poplar [4, 5]. The enzyme catalyses the conversion of dimethylallyl diphosphate (DMAPP) to isoprene by elimination of pyrophosphate in a divalent cation-dependent reaction [6]. *Bacillus* species are found to be the highest producers of isoprene among bacteria [3], however, the enzyme responsible for isoprene synthesis has not been identified. Previous reports revealed that *Bacillus subtilis* contained an isoprene synthase activity that catalyses DMAPP-dependent isoprene formation, but the activity was very labile [7].

There are two natural pathways for biosynthesis of DMAPP which is the precursor of isoprene: the mevalonic acid (MVA) pathway and the methylerythritol 4-phosphate (MEP) pathway [8–11]. The last enzyme in the MEP pathway is 4-hydroxy-3-methyl-butenyl diphosphate reductase (IspH), which contains a [4Fe−4S] cluster with a unique fourth iron not coordinated to any amino acid residue and which catalyses the reductive dehydration of (*E*)-4-hydroxy-3-methyl-2-butenyl diphosphate (HMBPP) to form an approximate 5:1 mixture of isopentenyl diphosphate (IPP) and DMAPP [12–16]. This conversion is a reductive process that requires a special electron transport system to provide two electrons to the substrate. In *Escherichia coli*, a system consisting of NADPH, flavodoxin, and flavodoxin reductase has been proposed to be the natural electron source for IspH [14, 15, 17]. Many studies in vitro have also demonstrated that *E. coli* IspH catalytic activity is detected in the presence of this system. IspH from the malaria parasite *Plasmodium falciparum,* which has no flavodoxin protein, uses ferredoxin (Fd) and ferredoxin-NADP^+^ reductase (FNR) as the reduction system [18].

**Fig. 1:**
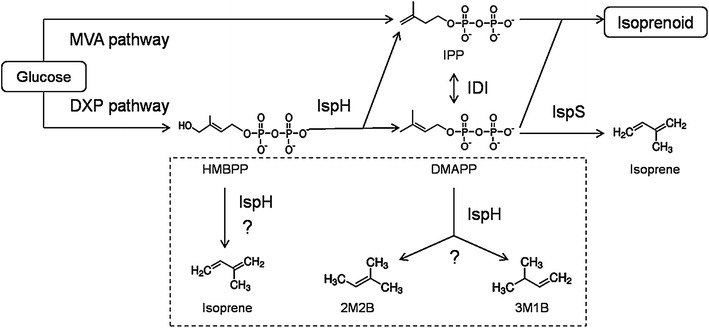
The mevalonic acid (MVA) pathway and the methylerythritol 4-phosphate (MEP) pathway for isoprenoid biosynthesis and two side reactions by IspH

Some substrate analogues are also converted by the IspH protein [19, 20]. In addition, IspH has been found to show acetylene hydratase activity, and catalyse the hydration of acetylenes to aldehydes and ketones [21]. These observations indicate that IspH is promiscuous in its catalytic activity. In this work, we revealed two further unexpected promiscuous reactions of IspH (Fig. 1), the formation of isoprene from HMBPP, and the conversion of DMAPP into a mixture of 2-methyl-2-butene (2M2B) and 3-methyl-1-butene (3M1B).

## Results

### Overexpression of *ispH* resulted in isoprene and isoamylene synthesis

We found that alkaliphilic *Bacillus* sp. N16-5 could produce a high level of isoprene; the maximum production reached 75 µg L^−1^ OD^−1^ (1 OD_600_ of 1 L bacterial culture produce 75 µg of isoprene). To identify the gene responsible for isoprene biosynthesis in the strain, a fosmid library was constructed and screened. The isoprene synthase gene was not found. However, a clone strain produced two unknown substances and a small amount of isoprene compared with other clones, as determined by gas chromatography (GC) analysis of the headspace of liquid cultures (Additional file 1: Figure S1). These two unknown substances were identified as 2M2B and 3M1B by GC–MS analysis (Additional file 1: Figure S2). After investigation of genes in DNA fragments that were ligated into the fosmid vector harboured by this clone, we concluded that the *ispH* gene encoding the IspH protein and the *fer* gene encoding a [4Fe−4S] type ferredoxin were involved in isoamylene synthesis.

Plasmid pSTV165H, encoding the *Bacillus* sp. N16-5 *ispH* gene, and plasmid pSTV165HF, encoding the *ispH* and *fer* genes, were constructed and transformed into the *E. coli* strain Trans109. *E. coli* Trans109 containing pSTV165HF could produce 2M2B and 3M1B, whereas the strain containing pSTV165H produced little isoamylene. This indicated that IspH was able to produce isoamylene in vivo in the presence of ferredoxin, which generally functions as an electron mediator in a variety of metabolic processes. To test whether the IspH proteins from *E. coli* and *B. subtilis* synthesised isoamylene, plasmids pSTV12HF (encoding *E. coli* K12 *ispH* and *fld*) and pSTV168HF (encoding *B. subtilisispH* and *fer*) were constructed and respectively transformed into *E. coli* Trans109. Both recombinant strains could produce isoamylene. The maximum production by *E. coli* containing pSTV12HF was 3.8 µg L^−1^ OD^−1^, compared with 7.6 µg L^−1^ OD^−1^ for *E. coli* containing pSTV168HF, and 9.2 µg L^−1^ OD^−1^ for *E. coli* containing pSTV165HF. These results indicated that the activity of IspH from different species might be different.

**Fig. 2:**
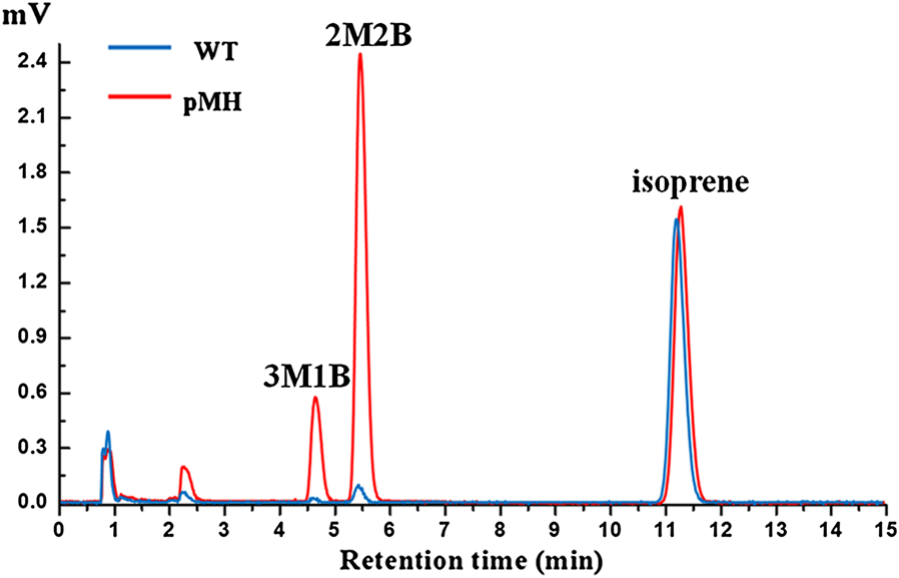
GC profile of headspace of *Bacillus* sp. N16-5 (wild-type) and engineered strain N16-5 containing the pMH plasmid with *ispH* gene after incubation at 37 °C for 12 h. The retention times of 3M1B, 2M2B and isoprene are 4.5, 5.5 and 11.3 min, respectively

Although *E. coli* containing pSTV165HF could produce 2M2B and 3M1B, *Bacillus* sp. N16-5 emitted little isoamylene. This may be because of the low concentration of IspH since there is only one copy of the *ispH* gene on the chromosome. To enhance IspH expression, plasmid pMH was constructed, harbouring the *ispH* gene under the control of the promoter of the *Bacillus* sp. N16-5 lactate dehydrogenase gene *ldh*, and transformed into *Bacillus* sp. N16-5. As expected, strain N16-5 containing the pMH plasmid produced up to 57.5 µg L^−1^ OD^−1^ of 2M2B and 11.6 µg L^−1^ OD^−1^ of 3M1B, much more than the wild-type strain (Fig. 2). However, the isoprene production of strain N16-5 remained the same. Notably, the ratio of 2M2B and 3M1B was approximately 5:1. Interestingly, the ratio was similar to that of IPP and DMAPP produced by IspH.

### Expression and purification of recombinant proteins

**Fig. 3:**
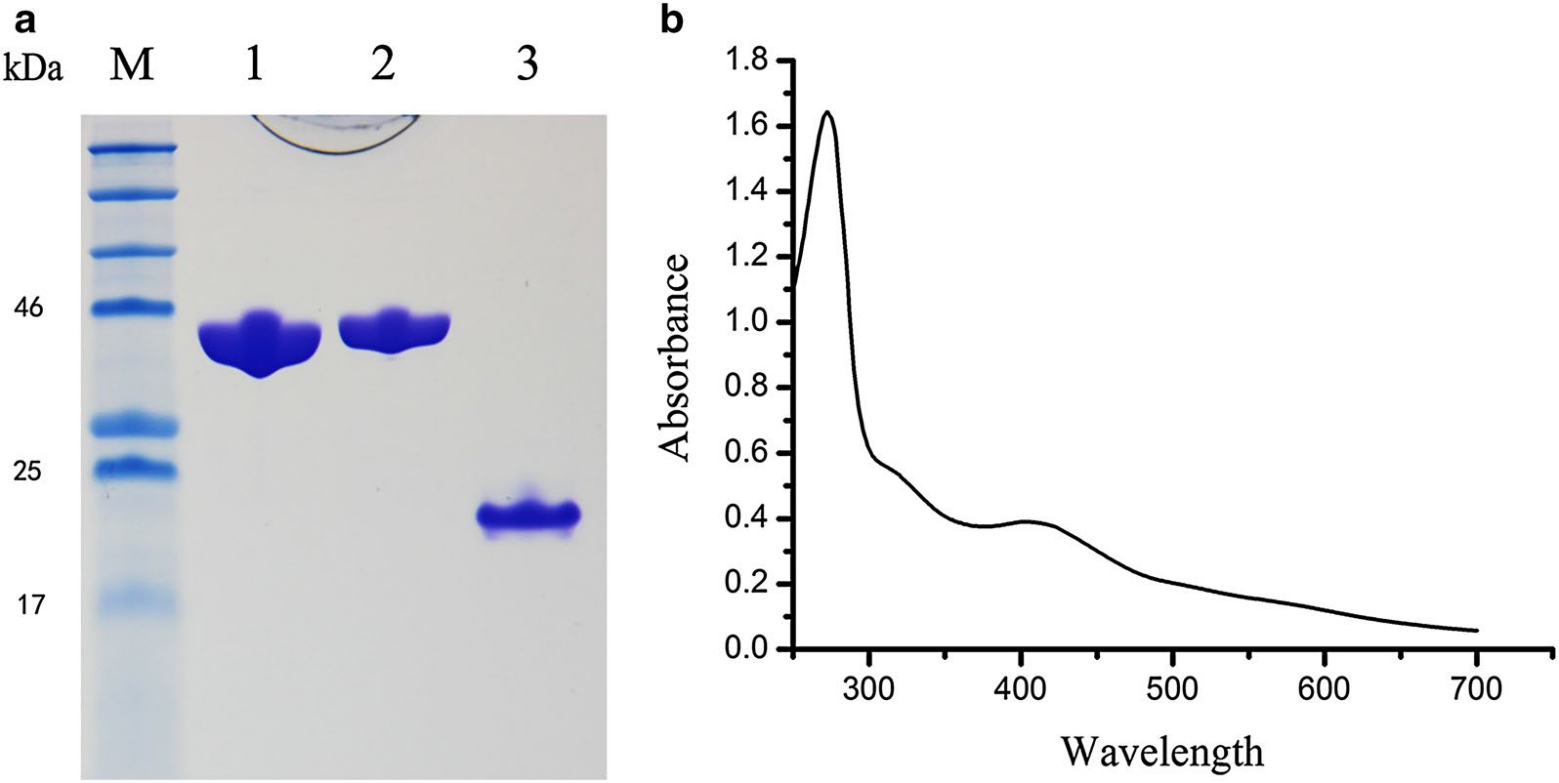
SDS polyacrylamide gel electrophoresis and UV–Vis spectrum of recombinant IspH protein. **a** SDS-PAGE: *Lane M*, molecular weight markers; *lane 1*, FNR protein; *lane 2*, IspH; *lane 3*, ferredoxin. **b** UV–Vis spectrum of recombinant IspH (1.5 mg mL^−1^) recorded in 20 mM Tris–HCl, pH 7.8. The spectrum shows a maximum at 410 nm and a shoulder at 320, indicating the presence of an iron–sulphur cluster

The aforementioned results implied that an unknown substance might be converted into isoamylene in a reaction catalysed by IspH in presence of ferredoxin. To test this possibility, IspH protein, ferredoxin and ferredoxin-NADP^+^-reductase were each expressed with a C-terminal His-tag. These recombinant proteins were directly used for an enzyme activity assay. Notably, IspH contains an iron–sulfur cluster, which may be crucial for catalytic activity and must be assembled by special biochemical machinery. Therefore, to improve the production of fully functional IspH, we used an iron–sulfur cluster biosynthesis hyperexpression strain, which has been described previously [15]. The purified recombinant IspH protein was intensely brown and appeared homogeneous after elution judged by sodium dodecyl sulphate (SDS) polyacrylamide gel electrophoresis (Fig. 3a). The absorption spectrum of the recombinant IspH had typical characteristics of iron–sulfur proteins with a maximum at approximately 410 nm, a shoulder at approximately 320 nm, and a peak at approximately 280 nm. The ratio of *E*_410_/*E*_280_ was 0.3 (Fig. 3b).

### Isoprene and isoamylene production by IspH in vitro

To assess whether purified recombinant IspH protein catalyses the formation of isoamylene from its substrate, IspH was incubated with NADPH, Fd, and FNR, which might be the in vivo IspH reducing system, and each of three potential substrates, HMBPP, IPP and DMAPP, in 5-mL sealed chromatography vials in anaerobic conditions (Table 1). The assay mixture without recombinant IspH was used as a negative control. The vial headspace was detected by GC–MS to identify isoamylene and isoprene. DMAPP could be converted to 2M2B and 3M1B by IspH in the presence of the NADPH-Fd-FNR reductant system (Table 1). There was a small amount of isoprene in the headspace of the vial containing DMAPP, which did not increase significantly compared with the negative control. The product ratio of 2M2B to 3M1B was approximately 8.5:1, which was different from the 5:1 ratio observed in vivo. When HMBPP was used as substrate of the recombinant IspH, isoprene was, surprisingly, produced in the vial headspace, while no isoprene was detected in the negative control. As the reaction proceeded, 2M2B and 3M1B were also detected in the headspace. We suggest that the DMAPP originating from HMBPP was further converted to isoamylene, but the isoprene in the vial headspace was directly derived from HMBPP by IspH. IPP was not converted to isoamylene or isoprene by IspH in our conditions.

**Table 1:** Enzyme tests with IspH

Substrate	Products in the headspace (ng/mL)			[2M2B]:[3M1B]
	Isoprene	2M2B	3M1B	
DMAPP	+^a^	39.1 ± 3.9	4.58 ± 0.6	8.5:1
IPP	–	–	–	
HMBPP	21.6 ± 2.3	6.23 ± 1.1	0.83 ± 0.1	7.5:1

Values are presented as the average ± standard deviation of three independent experiments

The reaction was performed in 20 mM Tris–HCl buffer (pH 7.8) containing 150 mM NaCl at 37 °C for 1 h

^a^Trace amount of isoprene, as was also observed in the negative control

A series of tests were performed in anaerobic conditions to confirm that each component of the reaction system was essential for the conversion of DMAPP/HMBPP (Additional file 1: Table S1). The results demonstrated that the recombinant IspH protein that catalysed the formation of isoamylene and isoprene required ferredoxin and FNR proteins in addition to NADPH as a cofactor. *Bacillus* sp. N16-5 has a flavodoxin that might be another mobile electron carrier; however, when it was substituted for Fd in the test, DMAPP and HMBPP could not be converted to isoamylene and isoprene by IspH. The addition of Mg^2+^ was not required for IspH activity in isoamylene and isoprene synthesis. To determine the kinetic parameters, IspH activity toward DMAPP was measured with DMAPP concentrations from 0.1 to 1 mM (Additional file 1: Figure S3). Based on Michaelis–Menten and Lineweaver–Burk plots, the DMAPP *K*_M_ was calculated to be approximately 275 µM. The maximal catalytic activity was approximately 6.2 nmol min^−1^ mg^−1^. It was difficult to determine the activity of IspH with HMBPP for isoprene formation because HMBPP was mostly transformed into IPP and DMAPP, and the latter could be further converted to isoamylene by IspH. In our test, the maximal activity obtained for the formation of isoprene from HMBPP was 2.9 nmol min^−1^ mg^−1^ in the presence of the NADPH-Fd-FNR reductant system. The recombinant IspH could also use reduced methyl viologen as an artificial electron donor, and the activity of IspH for isoprene formation was 3.5 nmol min^−1^ mg^−1^, slightly higher than that of IspH using its putative natural reducing system.

### Enzymatic activity assays in D_2_O

**Fig. 4:**
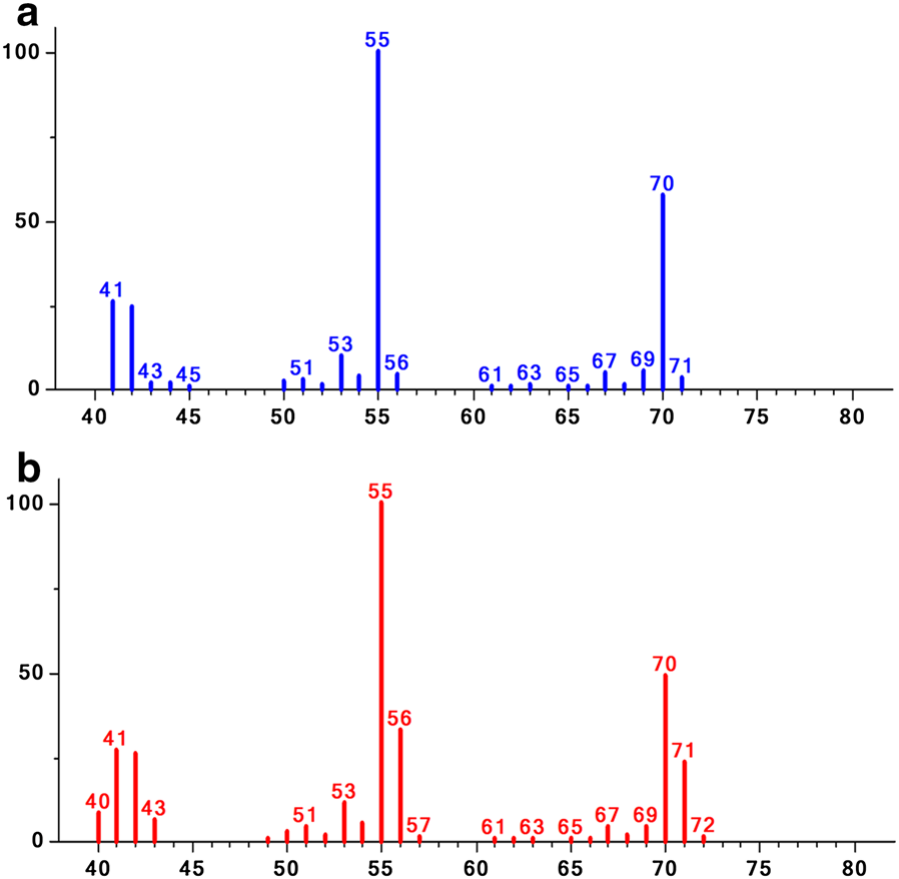
Mass spectrum of isoamylene. **a** Mass spectrum of 2M2B produced by the reaction that was performed in H_2_O buffer. **b** Mass spectrum of 2M2B produced by the reaction that was performed in 80 % (v/v) D_2_O buffer

To investigate proton transfer in these two reactions catalysed by IspH, enzymatic activity assays were performed in 80 % (v/v) D_2_O buffer and the products were identified by GC–MS. Protons are likely to derive from the bulk water of the reaction system; if the reaction is performed in D_2_O buffer, the molecular weight of the products of IspH would be increased, and the major peaks in the mass spectra would differ from those in H_2_O buffer. As expected, the mass spectrum showed that the ratios of fragment ions 71/70 and 56/55 of 2M2B produced by IspH in D_2_O buffer were significantly enhanced compared with the results in H_2_O buffer (Fig. 4); this fact suggested that the pyrophosphate group of DMAPP was eliminated and a proton from the bulk water could be used to form deuterium-2M2B. However, the abundance of fragment ion 70 was still greater than that of 71 in the mass spectrum, which was not consistent with the prediction based on the H_2_O/D_2_O ratio (1:4). In contrast to isoamylene, the mass spectrum of isoprene produced by IspH in D_2_O buffer remained unchanged, which demonstrated that C4 and C1 of HMBPP did not accept a proton after loss of the hydroxyl and pyrophosphate groups.

### Role of ferredoxin and flavodoxin in isoamylene and isoprene synthesis in vivo

**Fig. 5:**
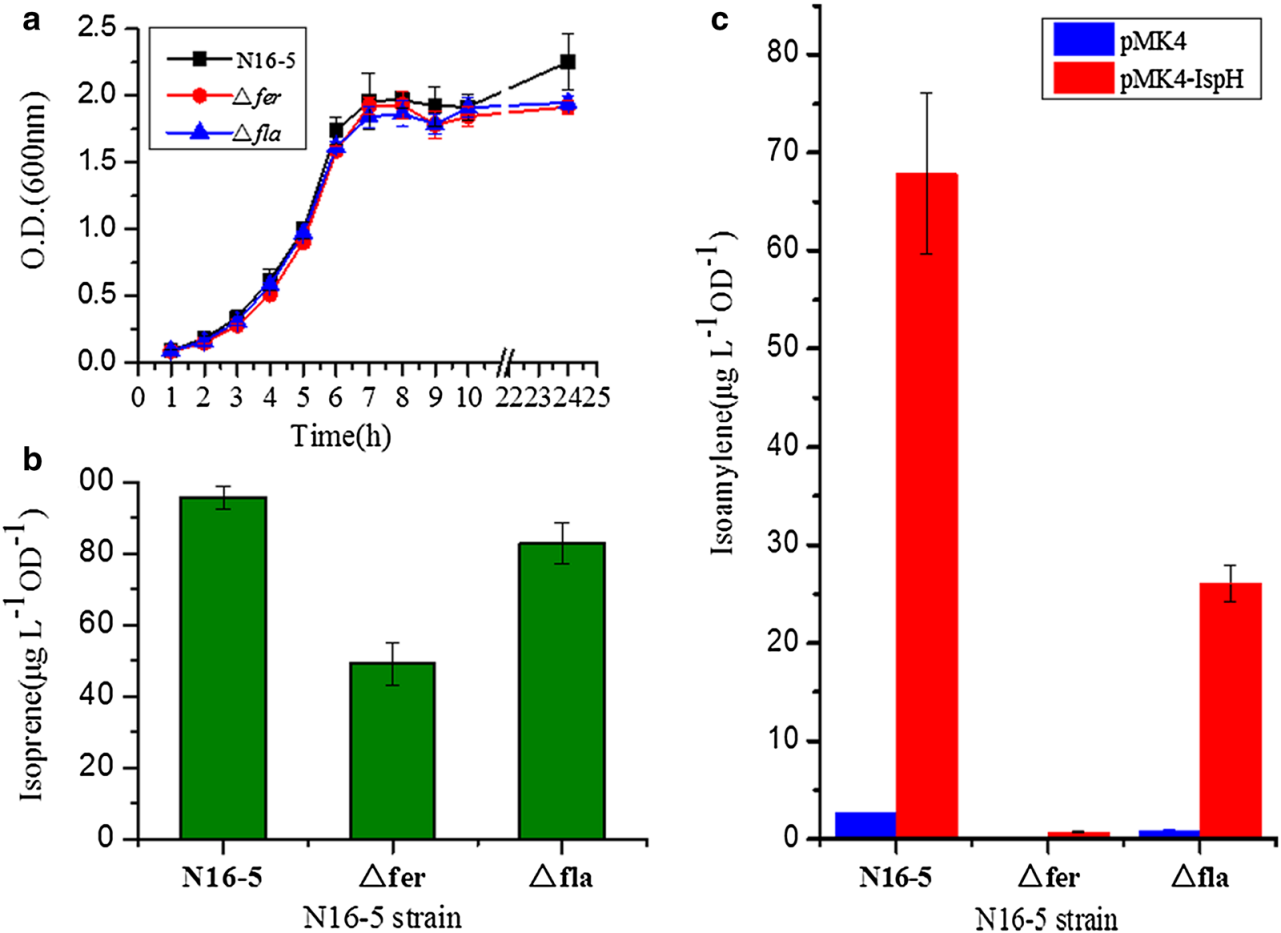
Growth phenotype and isoprene and isoamylene production by *Bacillus* sp. N16-5 and its derivatives. **a** *Bacillus* sp. N16-5 (wild-type), N165D1 (Δ*fer*) and N165D2 (Δ*fld*) strains were grown in Horikoshi-I medium at 37 °C on a rotary shaker at 220 rpm. **b** Isoprene production by these strains. **c** Isoamylene production by these strains when containing pMK4 or pMH plasmid with *ispH* gene. *Error bars* represent the standard deviations of three replicates

*Bacillus* sp. N16-5 has one [4Fe−4S] type ferredoxin and one flavodoxin. To evaluate whether they are required for isoamylene and isoprene synthesis in vivo, we constructed strain N165D1, which is a *fer* (ferredoxin) gene knockout, and strain N165D2, which is a *fld* (flavodoxin) gene knockout. Both mutant strains grew almost normally in Horikoshi-I medium (Fig. 5a). This result suggests that ferredoxin and flavodoxin are not essential for IspH activity in *Bacillus* sp. N16-5, which utilises the MEP isoprenoid biosynthesis pathway to produce IPP and DMAPP, two universal precursors of terpenes. However, the production of isoprene emitted by strain N165D1 was reduced by approximately 50 % compared with the wild-type strain. In contrast, isoprene production by strain N165D2 was close to that of the wild-type strain (Fig. 5b). Because IspH overexpression in *Bacillus* sp. N16-5 resulted in much more isoamylene production (see above), plasmid pMH, containing the *ispH* gene, was transformed into the mutant strains N165D1 and N165D2. Strain N165D1 harbouring pMH produced little isoamylene, while strain N165D2 harbouring pMH produced 32 µg L^−1^ OD^−1^ isoamylene, which was approximately half the level of the wild-type strain harbouring pMH (Fig. 5c). These results indicated that the absence of ferredoxin not only reduced isoprene production by half but nearly blocked isoamylene synthesis in *Bacillus* sp. N16-5. Combined with our in vitro enzymatic activity assay results, these findings demonstrate that ferredoxin is more efficient than flavodoxin as an electron donor to IspH in *Bacillus* sp. N16-5.

### The roles of IspH protein residues in the promiscuous activities

Based on previous reports, there are several highly conserved amino acid residues that contribute to IspH activity toward HMBPP [22–25]. To investigate the roles of these conserved amino acids in the promiscuous activities of IspH, the corresponding IspH variants were constructed. As described above, *E. coli* Trans109 harbouring plasmid pSTV165HF, used for coexpression of the *Bacillus* sp. N16-5 *ispH* and *fer* genes, could produce isoamylene and isoprene; therefore, IspH variants activity could be determined by quantitative analysis of isoamylene and isoprene produced by recombinant strains containing protein variants. Replacement of the strictly conserved His131 (H124 in *E. coli* IspH) by Asn, or Glu133 (E126 in *E. coli* IspH) by Gln, led to loss of isoamylene synthesis activity (Additional file 1: Figure S4), but both IspH variants could still produce isoprene.

For confirmation of these results, in vitro enzymatic activity assays were performed in anaerobic conditions. The H131N and E133Q IspH variant proteins were expressed and purified. The purified recombinant IspH variants were incubated with DMAPP or HMBPP in the presence of the NADPH-Fd-FNR reductant system in 5-mL sealed chromatography vials. DMAPP was not converted to isoamylene by either the H131N or E133Q IspH variant proteins, however, both could catalyse the formation of isoprene from HMBPP; the maximal activities of the H131N and E133Q IspH variant proteins were 2.5 and 3.1 nmol min^−1^ mg^−1^, respectively. It was notable that IspH H131 and E133 are strictly conserved and play a crucial role in the activity toward HMBPP [23, 24, 26]. To examine whether the H131N and E133Q IspH variant proteins could catalyse the transformation of HMBPP into DMAPP and IPP, their reaction products were analysed by an acid hydrolysis method, as described in previous reports [27, 28]. The two variant proteins lost the HMBPP reductase activity, which was consistent with previous reports [23, 25]. As a consequence, the H131N or E133Q IspH variants could be seen as isoprene synthases specialized for the conversion of HMBPP into isoprene.

**Fig. 6:**
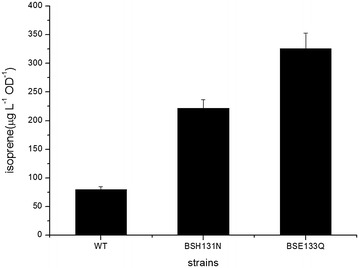
Isoprene production by *Bacillus* sp. N16-5 strains. *Error bars* represent the standard deviations of three replicates

To further investigate the activities of the H131N and E133Q IspH variants in *Bacillus* sp. N16-5, the corresponding pMH variants were obtained by site-directed mutagenesis and transformed into *Bacillus* sp. N16-5 to construct strains BSH131N and BSE133Q, respectively. After analysis of the headspace of cultures of these bacteria by GC–MS, we found that isoprene production by strain BSH131N was increased to three times the level of that by the wild-type strain, i.e. up to 235 µg L^−1^ OD^−1^; isoprene emitted by the BSE133Q strain reached approximately 352 µg L^−1^ OD^−1^ (Fig. 6). Neither strain emitted isoamylene. These results suggest that the H131 N and E133Q IspH variant proteins can be used for isoprene production.

## Discussion

Over the years, IspH, the last enzyme in the MEP pathway, has received considerable attention because it is not present in humans but is essential for microorganisms and thus is a viable target for the development of new antimicrobial drugs [29–31]. The structure and reaction mechanisms of IspH have been extensively studied. Based on previous reports, IspH shows promiscuous activity toward acetylenes. It can convert but-3-ynyl diphosphate and pent-4-ynyl diphosphate into an aldehyde and a ketone respectively [21]. In the present work, we have determined that IspH catalyses two unexpected side reactions in addition to its main catalytic activity. One is the elimination of DMAPP pyrophosphate to form 2M2B and 3M1B. The ratio of the two isoamylenes produced by *Bacillus* sp. N16-5 IspH in vivo was approximately 5:1, while in in vitro enzymatic activity assays, the ratio was approximately 8.5:1. Interestingly, *E. coli* IspH also produced isoamylenes, but the in vivo assay demonstrated a 2M2B:3M1B ratio of approximately 1.5:1 (Additional file 1: Figure S5). The other side reaction is the formation of isoprene from HMBPP, which is used as substrate to produce IPP and DMAPP by IspH in the MEP pathway. To some extent, isoprene can be seen as a byproduct generated by IspH in the process of transformation of HMBPP into IPP and DMAPP.

**Fig. 7:**
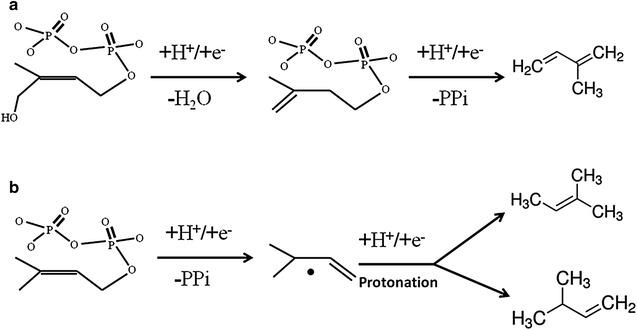
Potential mechanisms of isoprene and isoamylene formation by IspH. **a** Isoprene from HMBPP; **b** Isoamylene from DMAPP

The basis of these two reactions is that the pyrophosphate group of HMBPP or DMAPP can be eliminated by IspH. In fact, for HMBPP, PP_i_ is a better leaving group than an OH moiety, but conformational restrictions at the active site of IspH favour the OH group as the leaving group instead of pyrophosphate at the C1 position [23]. However, the formation of isoprene from HMBPP indicates that PP_i_ can be still removed with low activity by IspH. When the hydroxyl and pyrophosphate groups are all eliminated, the allyl intermediate forms, which would transform into isoprene instead of being protonated at the C1 and C4 positions; this proposed mechanism is supported by the data obtained in enzymatic activity assays in D_2_O buffer. In contrast, the formation of isoamylene from DMAPP would involve the introduction of a proton after the removal of PP_i_. Whether the isoamylene formed was 2M2B or 3M1B depends on the position of protonation (C1 or C3). Obviously, based on the product ratio of 2M2B to 3M1B, the protonation preferentially occurs at the C1 position. Based on our results, the two unexpected reactions catalysed by IspH are reductive processes requiring an electron transfer system. Their potential catalytic mechanisms are shown in Fig. 7. Isoprene formation from HMBPP may include dehydroxylation leading to an allyl radical intermediate, followed by the elimination of pyrophosphate and the formation of isoprene (Fig. 7a). Isoamylene formation from DMAPP may include the elimination of pyrophosphate leading to an allyl anion intermediate, which is protonated either at C1 or C3 to form 2M2B or 3M1B (Fig. 7b). The strictly conserved amino acids His131 and Glu133 of IspH may play crucial roles in the protonation, a conclusion supported by the fact that the H131N and E133Q IspH mutants could catalyse the elimination of both the hydroxyl and pyrophosphate groups of HMBPP to form isoprene, but could not catalyse isoamylene formation which involves the protonation step. Further studies are required to explore the catalytic mechanisms of the unexpected promiscuous activities.

Isoprene synthase (IspS) is the enzyme responsible for isoprene biosynthesis in plants. This enzyme catalyses the formation of isoprene by elimination of pyrophosphate from DMAPP. A number of studies of IspS for isoprene production have been reported [32–34]. Here we demonstrated that IspH, especially the H131N and E133Q variants, could be used for isoprene production. IspH uses HMBPP as substrate to form isoprene, which has two advantages over IspS. First, the accumulation of prenyl diphosphate can be avoided in the host cell. In previous study [35], prenyl diphosphate toxicity was observed in *E. coli* expressing a heterologous MVA pathway. Similarly, cytotoxicity related to prenyl diphosphate accumulation was reported in *B. subtilis* [36]. Second, the number of enzymatic reactions for isoprene production can be reduced because HMBPP can be directly converted into isoprene.

To date, the enzyme responsible for isoprene synthesis in bacteria, especially the *Bacillus* species which can produce more isoprene than others, has not been identified. Many attempts have been made to isolate IspS from *B. subtilis*, but without success [7, 37]. In this study, IspH was serendipitously found to catalyse the formation of isoprene from HMBPP. It is probable that IspH is responsible for isoprene production by bacteria. Isoprene may be a byproduct of IspH in the process of the transformation of HMBPP into IPP and DMAPP. Although, our results clearly demonstrate that IspH can catalyse isoprene formation from HMBPP, the existence of a specialized enzyme for isoprene synthesis in *Bacillus* sp. N16-5 cannot be ruled out. To investigate this question, we have attempted to disrupt the *ispH* gene in *Bacillus* sp. N16-5 in the presence of the MVA pathway from *Enterococcus faecalis*, but without success.

## Conclusions

We have revealed two unexpected side reactions of IspH for the first time. These new discoveries provide important insight into the IspH protein, which is a putative target for antimicrobial drugs for the treatment of malaria and tuberculosis. IspH, especially the H131 N and E133Q IspH variants can catalyse isoprene formation from HMBPP, which may be a good alternative to IspS from plants for isoprene production. In addition, it is most probable that the promiscuous activity of IspH is the cause of isoprene production by bacteria. More details of the promiscuous activity of IspH will be investigated in future study.

## Methods

### Bacterial strains and culture conditions

The bacterial strains and plasmids used in this study are listed in Additional file 1: Table S2. *E. coli* strains were routinely grown in Luria–Bertani (LB) broth at 37 °C on a rotary shaker at 220 rpm. For general purposes, *Bacillus* sp. N16-5 and its derivatives were grown aerobically in Horikoshi-I medium with 2 % NaCl (pH 10.0). To construct mutant strains, *Bacillus* sp. N16-5 and its derivatives were grown in complex neutral medium (NCM) (pH 7.7) or SA5 agar medium [38]. When necessary, antibiotics were added to the growth media at the following concentrations: ampicillin, 100 µg mL^−1^; kanamycin, 50 µg mL^−1^; chloramphenicol, 12.5 µg mL^−1^. For selection of *Bacillus* sp. N16-5 transformants, the final antibiotic concentrations were chloramphenicol at 2.5 µg mL^−1^ and erythromycin at 1 µg mL^−1^ in NCM or SA5 medium.

### Qualitative and quantitative analysis of isoamylene and isoprene

For identification of isoamylene, a gas chromatography-mass spectrometry (GC–MS) method was established as follows. An Agilent Technologies 7890B GC/5977A MSD that was equipped with an Agilent HP-PLOT Al_2_O_3_/S GC Column **(**25 m, 0.32 mm, 8.00 µm, 7-in. cage) with particle traps was used. Helium was used as the carrier gas at a flow rate of 2 mL min^−1^. The oven was kept at a constant temperature of 110 °C. To identify isoamylene production, peak retention times and mass spectra obtained from the bacterial headspace were compared with the retention time and mass spectrum of 2M2B and 3M1B standards (Sigma, St.Louis,MO, USA). For quantitative analysis of isoamylene production, bacterial strains were inoculated into headspace vials with Teflon-lined silicone septa at an inoculum concentration of 2 % and grown for 12 h at 37 °C on a rotary shaker at 220 rpm. The headspace of the medium was used as a negative control. A volume of 1 mL of headspace was sampled using a gas-tight syringe (SGE, Melbourne, Australia) and injected into the GC7800 GC inlet port (PuRui Instrument, Beijing, China), which had been equipped with a flame ionisation detector. The peak area was converted to isoamylene or isoprene concentration by comparing it with a standard curve that was plotted with a set of known concentrations of isoamylene or isoprene.

### Coexpression of *ispH* with *fer* in *E. coli*

For coexpression of the *Bacillus* sp. N16-5 *ispH* and *fer* genes, the plasmid pSTV165HF was constructed as follows. The complete *ispH* gene was amplified by PCR, and the product was cloned into vector pSTV28 (TaKaRa, Shiga, Japan) between *Hin*dIII and *Bam*HI sites, generating the plasmid pSTV165H. The *fer* gene was fused with the P_*lac*_ promoter and then ligated into pSTV165H at the *Eco*RI and *Bam*HI sites, to generate plasmid pSTV165HF. For coexpression of the *ispH* gene of *B. subtilis* 168 with the corresponding ferredoxin and the *ispH* gene of *E. coli* K12 with the corresponding flavodoxin, plasmids pSTV168HF and pSTV12HF were constructed as described above. These three constructs were transformed into *E. coli* strain Trans109, and transformants were selected on LB agar supplemented with 25 µg mL^−1^ chloramphenicol.

### Construction of mutant strains

To construct *Bacillus* sp. N16-5 mutant strains and the plasmids pND1 and pND2, derivations of the shuttle vector pNNB194 [39] were used as gene deletion constructs. Each plasmid, which contained approximately 1.6 kb of DNA homologous to the deletion-flanking region, was constructed as follows. Two sets of primers (Additional file 1: Table S3) were designed; one set amplified an 800-bp upstream fragment, which contained the 5ʹ region of each gene to be deleted, and the other amplified 800-bp of a downstream fragment, which contained the 3ʹ region of the target gene. The two fragments were fused together by overlap extension PCR, and the product was digested with *Eco*RI and *Bam*HI (NEB, Ipswich, USA) and ligated into the shuttle vector pNNB194, which had already been digested with the same enzymes. Finally, plasmids pND1 and pND2 were obtained. The deletion plasmids were transformed into *Bacillus* sp. N16-5 using the protoplast transformation procedure that was developed by Chenghua Gao [38], and transformants were selected on SA5 agar plates that had been supplemented with 1 µg mL^−1^ erythromycin at 34 °C. A single transformant was then inoculated into NCM medium, followed by double crossover and selection for the loss of the target gene from its locus. The final mutant clones were confirmed by PCR.

### Overexpression of *ispH* in *Bacillus* sp. N16-5

Plasmid pMH, which was used for *ispH* overexpression in *Bacillus* sp. N16-5 and its derivatives, was constructed as follows. The *ispH* gene was amplified by PCR with the primers listed in Additional file 1: Table S1 using N16-5 strain genomic DNA as the template. The PCR products were fused with the native *Bacillus* sp. N16-5 *ldh* promoter by overlap PCR, digested with *Eco*RI and *Bam*HI (NEB), and ligated into the corresponding site of the shuttle vector pMK4 [40] to create pMH. This plasmid with the *ispH* gene under transcriptional control of the P_*ldh*_ promoter was then transformed into *Bacillus* sp. N16-5 and the corresponding mutant strains using a protoplast transformation method [38].

### Expression and purification of recombinant proteins

For expression of IspH, ferredoxin and ferredoxin-NADP^+^-reductase from *Bacillus* sp. N16-5, their open reading frames were amplified by PCR, and the amplified fragments were cloned into the *Nco*I/*Xho*I sites of pET-28a(+) (TaKaRa, Shiga, Japan) to yield pET-ispH, pET-fer and pET-fnr, respectively, followed by transformation into *E. coli* BL21 (DE3) cells. To improve IspH protein-specific activity, a *ispH* hyperexpression strain was constructed using the method of Gräwert et al. [15]. Part of the *isc* operon from *E. coli* was amplified by PCR using high-fidelity DNA polymerase. The products were digested with *Kpn*I and *Pst*I and then ligated into the corresponding site of vector pSTV28 (TaKaRa, Shiga, Japan). The resulting plasmid pST-isc was transformed into *E. coli* BL21 (DE3) harbouring the pET-ispH plasmid. The recombinant strain was grown in LB medium supplemented with kanamycin (50 µg mL^−1^) and chloramphenicol (25 µg mL^−1^) at 37 °C. When the OD_600_ reached approximately 0.6, cysteine (1 mM), FeCl_3_ (0.1 mM) and IPTG (0.1 mM) were added to the culture, which was then incubated for 16 h at 18 °C on a rotary shaker at 110 rpm. The cells were harvested by centrifugation, washed twice with ddH_2_O, and stored at −80 °C.

Purification of recombinant proteins was performed in anaerobic conditions in a glovebox (Shel Lab, Cornelius, Oregon, USA) that had been flushed with a gas mixture of 95 % N_2_ and 5 % H_2_. Residual O_2_ was removed with palladium catalysts. Buffers were equilibrated overnight in the tent under stirring and were degassed by ultrasound before use. The harvested cells were resuspended and lysed in BugBuster Protein Extraction Reagent (Merck, Darmstadt, Germany) by pipetting or gentle vortexing for 20 min at room temperature and then centrifuged at 16,000×*g* for 20 min at 4 °C. The supernatant was applied to a Ni–NTA column that had been equilibrated with 20 mM Tris–HCl, pH 7.8, containing 5 mM imidazole and 150 mM NaCl. After washing with 20 mM imidazole, protein was eluted with 250 mM imidazole. Fractions were collected and desalted with a desalting column (Sangon Biotech, Shanghai, China). The purified protein was flash-frozen in liquid nitrogen and stored at −80 °C until use.

### Enzymatic activity assays

Enzyme-catalysed reactions were performed in 5-mL sealed chromatography vials in anaerobic conditions in a glovebox. Assay mixtures contained 150 mM NaCl, 20 mM Tris–HCl (pH 7.8), 2 mM DTT, 1 mM NADPH, 1 mM DMAPP (unless otherwise stated), 20 µM Fd, 5 µM FNR and 0.5 µM IspH. The total volume was 500 µL. To test the IspH protein substrate, DMAPP in the mixtures was replaced with IPP or HMBPP. After the mixtures were ready, the vial was immediately sealed and incubated at 37 °C for 1 h. Headspace gas (1 mL) was sampled with a needle and syringe. Headspace of the mixtures with no substrate was run as a negative control. To avoid creating a vacuum in the vial, 1 mL water was injected into the vial concurrent with the removal of the headspace.

### Site-directed mutagenesis

To investigate the roles of specific protein residues in catalytic activity, site-directed mutagenesis was performed using mismatch PCR as follows. The entire pSTV165HF plasmid was amplified by PCR using high-fidelity DNA polymerase with mutagenic primers. The amplification products were digested with *Dpn*I for 2 h at 37 °C and then transformed into competent *E. coli* strain DMT (TransGene Biotech, Beijing, China). Transformants were selected on LB-agar plates supplemented with 25 µg mL^−1^ erythromycin, and the final mutant clones were confirmed by sequencing. The mutant plasmids were extracted and transformed into *E. coli* Trans109.

## Additional file

10.1186/s12934-016-0476-9 This file consists of five supplemental figures and three supplemental tables.** Figure S1** and** Figure S2** present additional information on identification of 3-methyl-1-butene, 2-methyl-2-butene and isoprene.** Table S1** presents isoamylene and isoprene production in various conditions.** Figure S3** presents Michaelis–Menten plots of the activity of recombinant IspH toward DMAPP.** Figure S4** shows isoamylene production by recombinant strains expressing IspH variants.** Figure S5** presents GC profile of 3-methyl-1-butene and 2-methyl-2-butene produced.** Table S2** and** Table S3** list strains, plasmids and primers used in this study.

## Abbreviations

MEP: methylerythritol phosphate
MVA: mevalonic acid
HMBPP: (*E*)-4-hydroxy-3-methyl-2-butenyl diphosphate
IPP: isopentenyl diphosphate
DMAPP: dimethylallyl diphosphate
IspH: (*E*)-4-hydroxy-3-methyl-2-butenyl diphosphate reductase
IspS: isoprene synthase
2M2B: 2-methyl-2-butene
3M1B: 3-methyl-1-butene
FNR: ferredoxin-NADP^+^ reductase
Fd: ferredoxin
OD_600_: the optical density at 600 nm
