# Supplementary material for: Two unexpected promiscuous activities of the iron–sulfur protein IspH in production of isoprene and isoamylene
Source: Microb Cell Fact. 2016 May 11;15:79. doi: 10.1186/s12934-016-0476-9 (PMC4864966; doi:10.1186/s12934-016-0476-9)
Supplement: Supplementary file 1 — 10.1186/s12934-016-0476-9 This file consists of five supplemental figures and three supplemental tables. Figure S1 and Figure S2 present additional information on identification of 3-methyl-1-butene, 2-methyl-2-butene and isoprene. Table S1 presents isoamylene and isoprene production in various conditions. Figure S3 presents Michaelis–Menten plots of the activity of recombinant IspH toward DMAPP. Figure S4 shows isoamylene production by recombinant strains expressing IspH variants. Figure S5 presents GC profile of 3-methyl-1-butene and 2-methyl-2-butene produced. Table S2 and Table S3 list strains, plasmids and primers used in this study. [file 12934_2016_476_MOESM1_ESM.pdf]

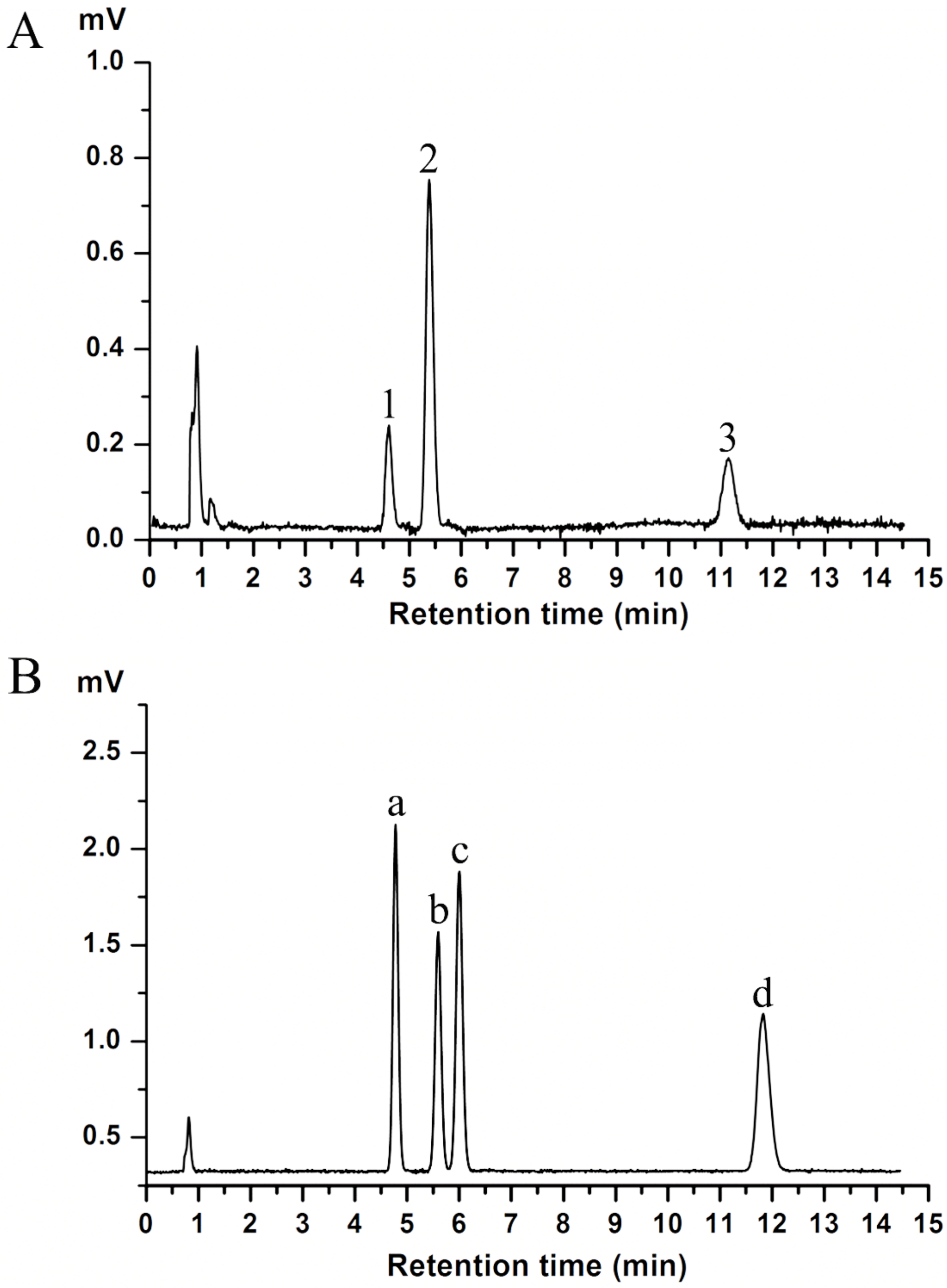


**Fig. S1 GC profile.** (A) GC profile of the headspace of the *E.coli* trans109 strain containing pSTV165HF after incubation for 10 h at 37℃. (B) GC profile of isoamylene and isoprene standard. (a) 3-methyl-1-butene; (b) 2-methyl-2-butene; (c) 2-methyl-1-butene; (d) isoprene.

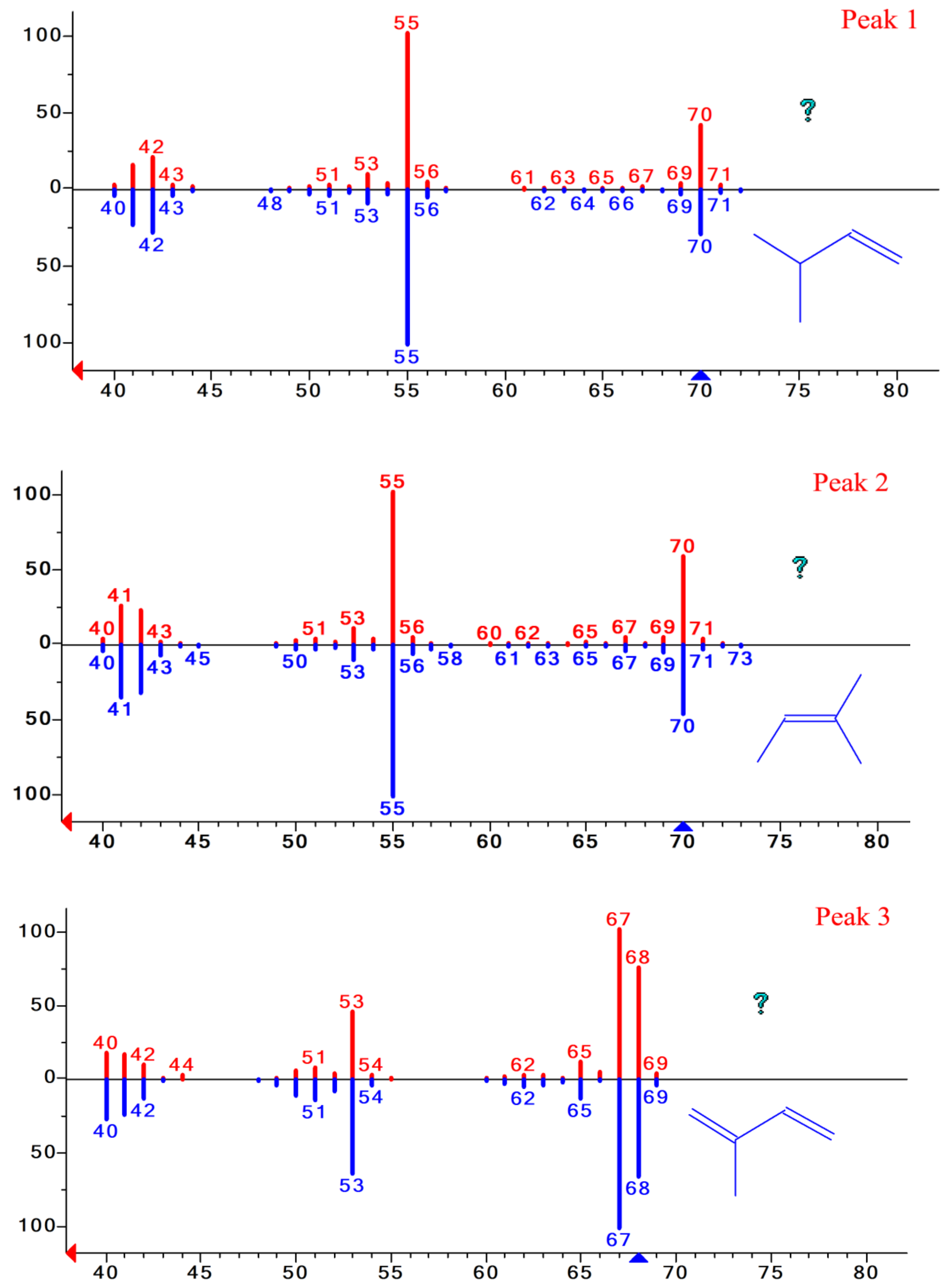


**Fig. S2.** Iditification of the unkown substances in the headspace of the liquid cultures by GC-MS analysis. Red is the mass spectrum of the sample, Blue is the mass spectrum of the standard. Peak 1, Peak 2 and Peak 3 are identified to be 3-methyl-1-butene, 2-methyl-2-butene and isoprene respectively.

Table S1. Isoamylene and isoprene reaction system production under various conditions

| Supplements | | | | | | Isoamylene/Isoprene production |
|---|---|---|---|---|---|---|
| IspH | FNR | Fdx | Fld | NADPH | $Mg^{2+}$ | |
| - | + | + | - | + | + | - |
| + | - | + | - | + | + | - |
| + | + | - | + | + | + | - |
| + | + | + | - | - | + | - |
| + | + | + | - | + | + | + |
| + | + | + | - | + | - | + |

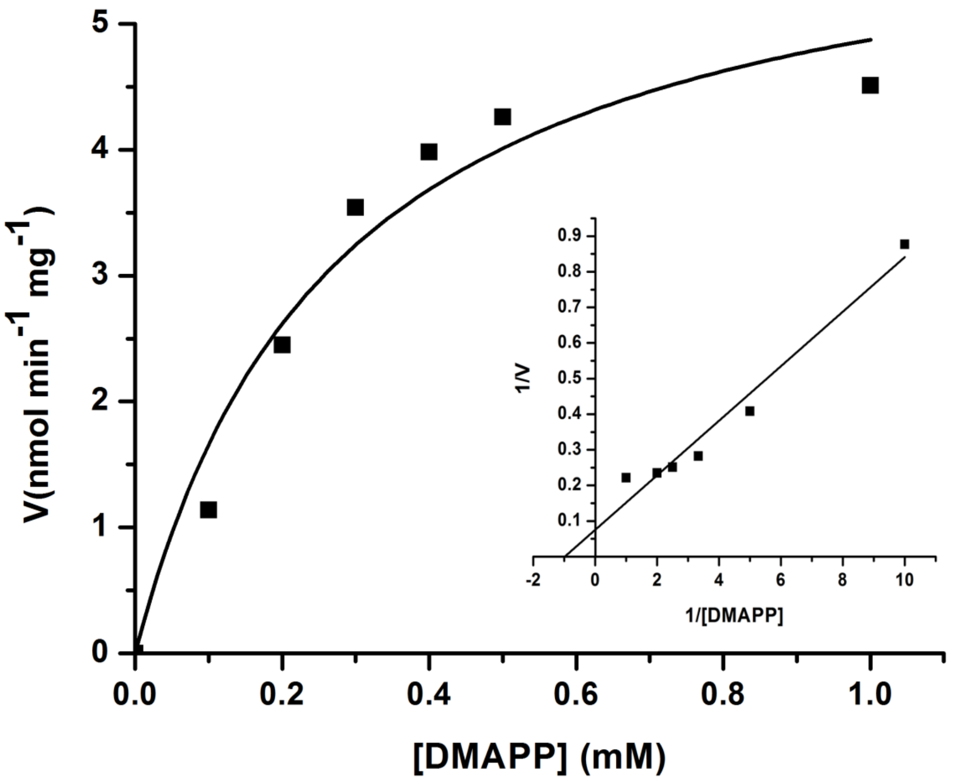


**Fig. S3.** Michaelis-Menten plot of the activity of the recombinant protein IspH toward DMAPP. DMAPP concentrations were varied from 0 to 1 mM. The *K*m value for DMAPP was approximately 275 μM. The inset shows the Lineweaver-Burk plot.

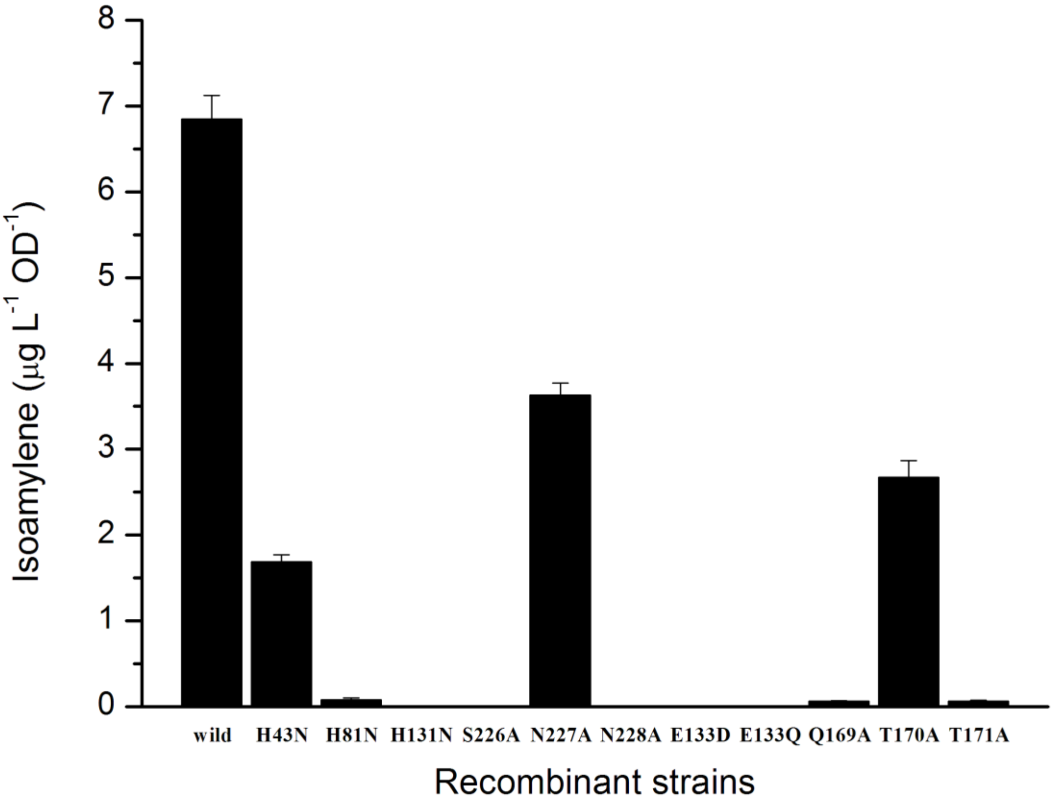


**Fig. S4.** Isoamylene production of the recombinant strains in which wild-type IspH and the IspH mutants were expressed. Error bars represent the standard deviations of three replicates.

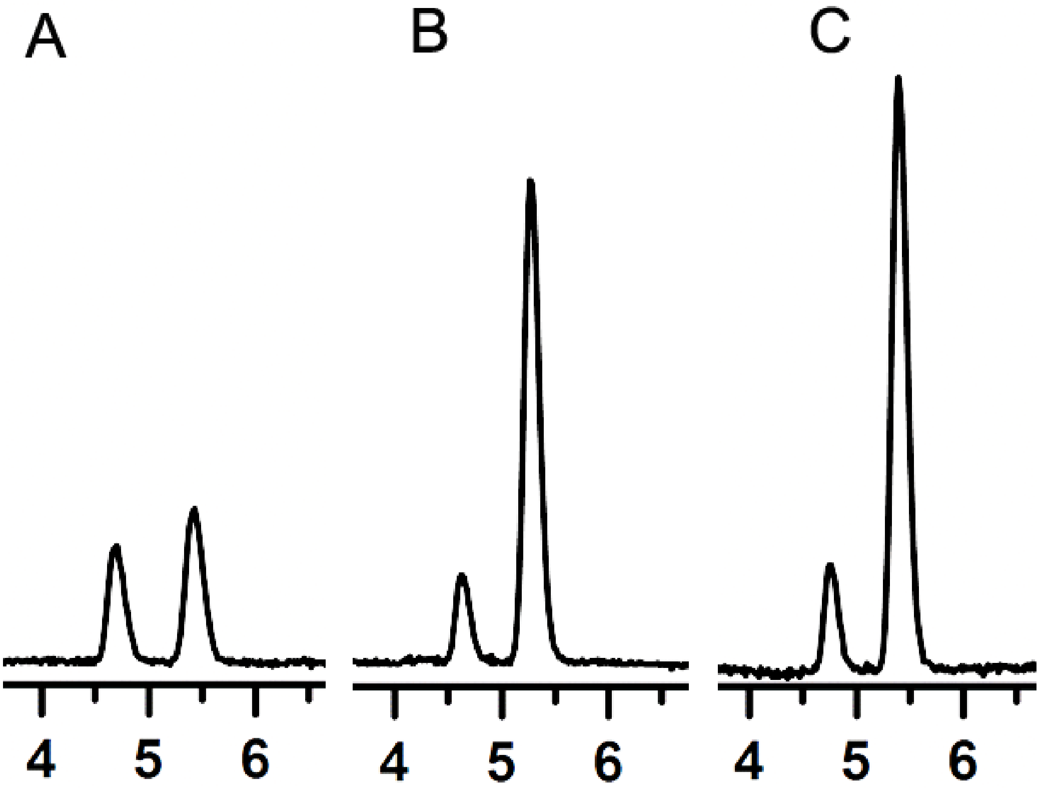


**Fig. S5.** GC profile of isoamylene (2M2B and 3M1B) produced by the recombinant *E.coli* strains. A. The production of *E.coli* trans109 strain containing pSTV12HF, the ratio of 2M2B (2.1 µg/L) and 3M1B (1.4 µg/L) is approximately 1.5:1. B. The production of of *E.coli* trans109 strain containing pSTV168HF, the ratio of 2M2B (6.1 µg/L) and 3M1B (1.2 µg/L) is approximately 5:1. C. The production of the *E.coli* trans109 strain containing pSTV165HF, the ratio of 2M2B (7.3 µg/L) and 3M1B (1.4 µg/L) is approximately 5:1.

**Table S2 Strains and plasmids in this study**

| Strain or plasmid | Genotype or relevant characteristic | Source or reference |
|---|---|---|
| ***E. coli*** | | |
| BL21(DE3) | F$^-$*ompT gal dcm lon hsdS*$_B$(*r*$_B^-$ *m*$_B^-$) *λ(DE3 [lacI lacUV5-T7gene1 ind1 sam7 nin5])* | TransGen Biotech |
| *Trans* DMT | F$^-$ *φ80 lacZΔM15 Δ(lacZYA-argF)U169 recA1 endA1 hsdR17(r*$_K^-$, *m*$_K^+$*) phoA supE44 thi-1 gyrA96 relA1 tonA* | TransGen Biotech |
| *Trans*109 | *endA1 recA1 gyrA96 thi-1 hsdR17 (r*$_k^-$*m*$_k^+$*)relA1 supE44 D (lac-proAB) [F'traD36 proAB laqI qZΔM15]* | TransGen Biotech |
| ***Bacillus*** | | |
| *Bacillus* sp. N16-5 | A facultative alkaliphilic strain, producing isoprene | Laboratory stock |
| N165D1 | Δ*fer* | This study |
| N165D2 | Δ*fld* | This study |
| N165H | *Bacillus* sp. N16-5 containing pMH | This study |
| N165D1H | N165D1 containing pMH | This study |
| N165D2H | N165D2 containing pMH | This study |
| *Bacillus subtilis* 168 | used for *ispH* and *fer* gene amplification | Laboratory stock |
| **Plasmid** | | |
| pSTV28 | *E. coli* cloning vector; Cm$^r$ | TaKaRa |
| pSTV165H | pSTV28 containing the *ispH* gene from *Bacillus* sp. N16-5 | This study |
| pSTV165HF | pSTV28 containing the *fer* gene and the *ispH* gene from *Bacillus sp.* N16-5 | This study |
| pSTV168HF | pSTV28 containing the *fer* gene and the *ispH* gene from *Bacillus subtilis* 168 | This study |
| pSTV12HF | pSTV28 containing the *fld* gene and the *ispH* gene from *Escherichia coli* | This study |
| pNNB194 | Shuttle vector used for gene disruption; Amp$^r$Erm$^r$ | Laboratory stock |
| pND1 | pNNB194 containing a 1.6 kb fragment fused by two 800 bp homologous regions flanking the *fer* gene | This study |
| pND2 | pNNB194 containing a 1.6 kb fragment fused by two 800 bp homologous regions flanking the *fld* gene | This study |
| pMK4 | Shuttle vector used for gene expression; Amp$^r$Cm$^r$ | Laboratory stock |
| pMH | pMK4 containing the *ispH* gene under the control of the $P_{ldh}$ promoter | This study |
| pET-28a(+) | used for expression of IspH, FNR and ferredoxin proteins from *Bacillus* sp. N16-5 | Novagen |

**Table S3 Primers used in this study**

| Primer | Sequence (5 '→3 ')[a] | Purpose |
|---|---|---|
| QFLAupF | CGGGATCCATCACGTGATCATATTATTGC | pND1 construction |
| QFLAupR | TCGTTATTTTAAGGTAGTAGCGCTTGCAAATACGAGTAATAC | pND1 construction |
| QFLAdownF | GTATTACTCGTATTTGCAAGCGCTACTACCTTAAAATAACGA | pND1 construction |
| QFLAdownR | CGGAATTCGATCAAGTTATGAAGCTTTCACG | pND1 construction |
| QFLAF | CTATTTCCTTGCTGTTTCCGATGGC | excision confirmation |
| QFLAR | ACATGAGGAGGTAGTTGTTAATGG | excision confirmation |
| QFERupF | CGGAATTCCTGTAGCCTGTGCTGAATGATG | pND1 construction |
| QFERupR | ACCGGTGGCCGATTATTTACACAAAAGCCTCCCATTAAAG | pND1 construction |
| QFERdownF | CTTTAATGGGAGGCTTTTGTGTAAATAATCGGCCACCGGT | pND1 construction |
| QFERdownR | CGGGATCCTCAATCAAATGTCATATAAACG | pND1 construction |
| QFERF | CCATAATCGATACATTACCGTG | excision confirmation |
| QFERR | CATGGTCCATCTCCTAACGTGC | excision confirmation |
| MPldhupF | CCGGAATTCCTGATGGTAGGACGCTTGTA | pMH construction |
| MPldh275F | GGAAATTTTCAAAACCTCCATGTTTAAACATCTACCTTTCCC | pMH construction |
| M275PldhR | GGGAAAGGTAGATGTTTAAACATGGAGGTTTTGAAAATTTCC | pMH construction |
| M275downR | CGGGATCCTTAGGCCCGCTCCGCTTTTTTCTTTCTC | pMH construction |

[a] Underlined, restriction enzyme recognition sites.
